# Complete genome of *Pieris rapae*, a resilient alien, a cabbage pest, and a source of anti-cancer proteins

**DOI:** 10.12688/f1000research.9765.1

**Published:** 2016-11-03

**Authors:** Jinhui Shen, Qian Cong, Lisa N. Kinch, Dominika Borek, Zbyszek Otwinowski, Nick V. Grishin

**Affiliations:** 1 Departments of Biophysics and Biochemistry, University of Texas Southwestern Medical Center, Dallas, USA; 2 Howard Hughes Medical Institute, University of Texas Southwestern Medical Center, Dallas, USA

**Keywords:** Butterfly genomics, Invasive species, Crop pest, Pieridae, Population history

## Abstract

The Small Cabbage White ( *Pieris rapae*) is originally a Eurasian butterfly. Being accidentally introduced into North America, Australia, and New Zealand a century or more ago, it spread throughout the continents and rapidly established as one of the most abundant butterfly species. Although it is a serious pest of cabbage and other mustard family plants with its caterpillars reducing crops to stems, it is also a source of pierisin, a protein unique to the Whites that shows cytotoxicity to cancer cells. To better understand the unusual biology of this omnipresent agriculturally and medically important butterfly, we sequenced and annotated the complete genome from USA specimens. At 246 Mbp, it is among the smallest Lepidoptera genomes reported to date. While 1.5% positions in the genome are heterozygous, they are distributed highly non-randomly along the scaffolds, and nearly 20% of longer than 1000 base-pair segments are SNP-free (median length: 38000 bp). Computational simulations of population evolutionary history suggest that American populations started from a very small number of introduced individuals, possibly a single fertilized female, which is in agreement with historical literature. Comparison to other Lepidoptera genomes reveals several unique families of proteins that may contribute to the unusual resilience of *Pieris*. The nitrile-specifier proteins divert the plant defense chemicals to non-toxic products. The apoptosis-inducing pierisins could offer a defense mechanism against parasitic wasps. While only two pierisins from *Pieris rapae* were characterized before, the genome sequence revealed eight, offering additional candidates as anti-cancer drugs. The reference genome we obtained lays the foundation for future studies of the Cabbage White and other Pieridae species.

## Introduction

**Figure 1.**
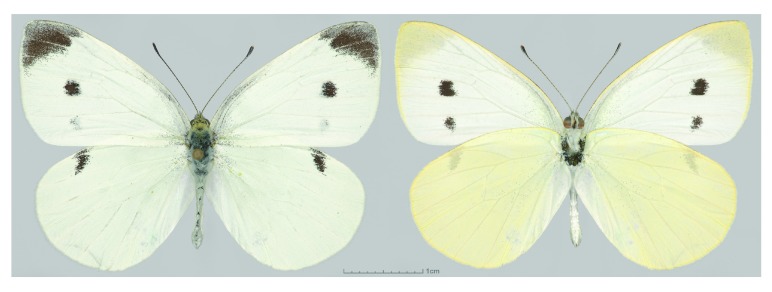
*Pieris rapae* specimen used for the paired-end genomic library constructions. Dorsal (left) and ventral (right) views are shown. Voucher NVG-4113, male, USA: Texas: Dallas Co., Dallas, GPS 32.90516, -96.81546, 17-Jul-2015.

The Small Cabbage White ( *Pieris rapae*, Figure 1), also known as European Cabbage Butterfly, or Imported Cabbageworm, is one of the most common and widely spread butterflies in North America, ranging from Southern Canada to Mexico ^1^. While mostly present in disturbed open habitats, it also invades valley bottoms, mountain tops, and forested areas ^2^. In many northeastern USA states, it frequently outnumbers all other butterflies combined ^3^. North American populations of the Cabbage Whites, currently numbering in billions, are likely a progeny of a single female accidentally introduced to Quebec, Canada during the second half of the 19 ^th^ century ^4, 5^. By the beginning of the 20 ^th^ century it had reached California Coast ^6^. Around the same time, it was introduced into Hawaii, New Zealand and Australia ^6, 7^. Originally from Eurasia and Northern Africa ^1^, Cabbage White has become one of the most ubiquitous butterfly species. The reasons for its population expansion across variable habitats as well as the population history of American invasion are poorly understood.

While only very few butterflies are agricultural pests, the Small White is notorious for reducing cabbage plants to stems. Going through its life-cycle quickly and having up to 6 generations per year ^8^, it is a serious pest of the mustard family crops ^5, 9^. In addition to damaging plants, caterpillars contaminate and stain produce with feces.

These butterflies are also a source of a protein with anti-cancer properties ^10^. Aptly termed pierisin, this enzyme of a probable bacterial origin is unique to *Pieris* and its close relatives among Lepidoptera species ^10, 11^. Pierisin contains an N-terminal ADP-ribosylation catalytic domain followed by four ricin domains, and it can induce apoptosis and thus contribute to metamorphosis and resistance to parasitoids ^11, 12^. Due to its cytotoxic effects on many cancer cell lines, pierisin is an unexpected protein of medical importance ^10^. Agricultural and medical significance of the Cabbage White has attracted broad attention from researchers and the general public. However, the lack of complete genome sequence hinders these studies.

To aid genetics, evolutionary, and biochemical studies of the Cabbage White, we sequenced and annotated its complete genome from North American specimens. At 246 Mbp, it is one of the smallest genomes among Lepidoptera genomes assembled to this day, and the first representative from the Pierinae subfamily. Overall, this diploid genome contains 1.5% heterozygous positions that is consistent with the expected high level of butterfly’s heterozygosity. However, the *Pieris* genome contains a large number of SNP-free segments that are at least 1000 bp long (with the median length equal to 38000 bp), which together constitute 18.3% of the assembled genome. This number is below 4% in other species. The high fraction of homozygous segments indicates low genetic diversity of the population, which supports the hypothesis that Cabbage White expansion in America started from a very small number of individuals, which could be as low as 1 or 2 fertilized females.

Comparison to other Lepidoptera genomes reveals several unique families of proteins that may contribute to the unusual resilience and adaptability of *Pieris*. For instance, the nitrile-specifier proteins, which converts plant defense chemicals to non-toxic molecules ^13^ are unique to these species. The apoptosis-inducing pierisins could offer a defense mechanism against parasitic wasps. While only two pierisins from *Pieris rapae* were characterized before ^14, 15^, the genome sequencing revealed eight genes coding for pierisins, offering additional candidates for anti-cancer drugs development. The reference genome we obtained lays the foundation for future studies of the Cabbage White and other species of Pieridae.

## Results and discussion

### Genome assembly, annotation, and comparison to other Lepidoptera genomes

We assembled a 246 Mb reference genome of *Pieris rapae* ( *Pra*), which is one of the smallest among currently sequenced Lepidoptera genomes ( Supplementary Table S1A) ^16– 26^. The scaffold N50 of *Pra* genome assembly is 617 kb, better than many other published Lepidoptera genomes ( Table 1). The genome assembly is also better than many other Lepidoptera genomes in terms of completeness measured by the presence of Core Eukaryotic Genes Mapping Approach (CEGMA) genes ( Supplementary Table S1B) ^27^, cytoplasmic ribosomal proteins and independently assembled transcripts ( Table 1). The genome sequence has been deposited at DDBJ/EMBL/GenBank under the accession LWME00000000. The version described in this paper is version LWME01000000. In addition, the main results from genome assembly, annotation and analysis can be downloaded at http://prodata.swmed.edu/LepDB/.

**Table 1.** Quality and composition of Lepidoptera genomes.

Feature	*Pra*	*Pse*	*Pgl*	*Ppo*	*Pxu*	*Dpl*	*Hme*	*Mci*	*Cce*	*Lac*	*Mse*	*Bmo*	*Pxy*
Genome size (Mb)	246	406	375	227	244	249	274	390	729	298	419	481	394
Genome size without gap (Mb)	243	347	361	218	238	242	270	361	689	290	400	432	387
Heterozygosity (%)	1.5	1.2	2.3	n.a.	n.a.	0.55	n.a.	n.a.	1.2	1.5	n.a.	n.a.	˜2
Scaffold N50 (kb)	617	257	231	3672	6199	716	194	119	233	525	664	3999	734
CEGMA (%)	99.6	99.3	99.6	99.3	99.6	99.6	98.2	98.9	100	99.3	99.8	99.6	98.7
CEGMA coverage by single scaffold (%)	88.7	87.4	86.9	85.8	88.8	87.4	86.5	79.2	85.3	86.8	86.4	86.8	84.1
Cytoplasmic Ribosomal Proteins (%)	98.9	98.9	98.9	98.9	97.8	98.9	94.6	94.6	98.9	98.9	98.9	98.9	93.5
*De novo* assembled transcripts (%)	99	97	98	n.a.	n.a.	96	n.a.	97	97	98	n.a.	98	83
GC content (%)	32.7	39.0	35.4	34.0	33.8	31.6	32.8	32.6	37.1	34.4	35.3	37.7	38.3
Repeat (%)	22.7	17.2	22.0	n.a.	n.a.	16.3	24.9	28.0	34.0	15.5	24.9	44.1	34.0
Exon (%)	7.9	6.20	5.07	5.11	8.59	8.40	6.38	6.36	3.11	6.96	5.34	4.03	6.35
Intron (%)	33.3	25.5	25.6	24.8	45.5	28.1	25.4	30.7	24.0	31.6	38.3	15.9	30.7
Number of proteins (thousands)	13.2	16.5	15.7	15.7	13.1	15.1	12.8	16.7	16.5	17.4	15.6	14.3	18.1
Number of universal ortholog lost	48	35	33	235	71	18	225	356	35	82	120	236	808
Number of species specific genes	27	101	32	9	240	69	52	59	101	87	165	98	399

*n.a.* Data not available

*Pra*: *Pieris rapae; Lac*: *Lerema accius; Cce*: *Calycopis cecrops*; *Pgl*: *Pterourus* glaucus; *Dpl*: *Danaus plexippus*; *Hme*: *Heliconius melpomene*; *Mci*: *Melitaea cinxia*; *Bmo*: *Bombyx mori*; *Pxy*: *Plutella xylostella*; *Mse: Manduca sexta*; *Ppo*: *Papilio polytes*; *Pse*: *Phoebis sennae*; *Pxu*: *Papilio xuthus*.

Heterozygosity: Calculated as the percent of heterozygous positions detected by the Genome Analysis Toolkit (GATK) for *Pgl*, *Lac*, *Cce*, *Pra* and *Pse*; or taken from information in the literature for *Dpl* ^20^; or estimated based on the histogram of K-mer frequencies for *Pxy* ^18, 41^.

We assembled the transcriptome of *Pra* using another specimen (NVG-3537) from the same locality. Based on the transcriptome, homologs from other Lepidoptera and *Drosophila melanogaster*, *de novo* gene predictions, and repeat identification ( Supplementary Table S2B), we predicted 13,188 protein-coding genes in the *Pra* genome ( Supplementary Table S2C). 74.4% of these genes are likely expressed in the adult, as they fully or partially overlap with the transcripts. We annotated the putative functions of the 10,747 protein-coding genes ( Supplementary Table S2D). Comparison of the protein sets from Lepidoptera species revealed the presence of some proteins unique to the Cabbage White and not present in other species. Among these are pierisins and nitrile-specifier proteins that play important roles in resistance against parasites and toxins from plants and contribute to the successful spread of *Pieris rapae* across continents.

### Phylogeny of Lepidoptera

**Figure 2.**
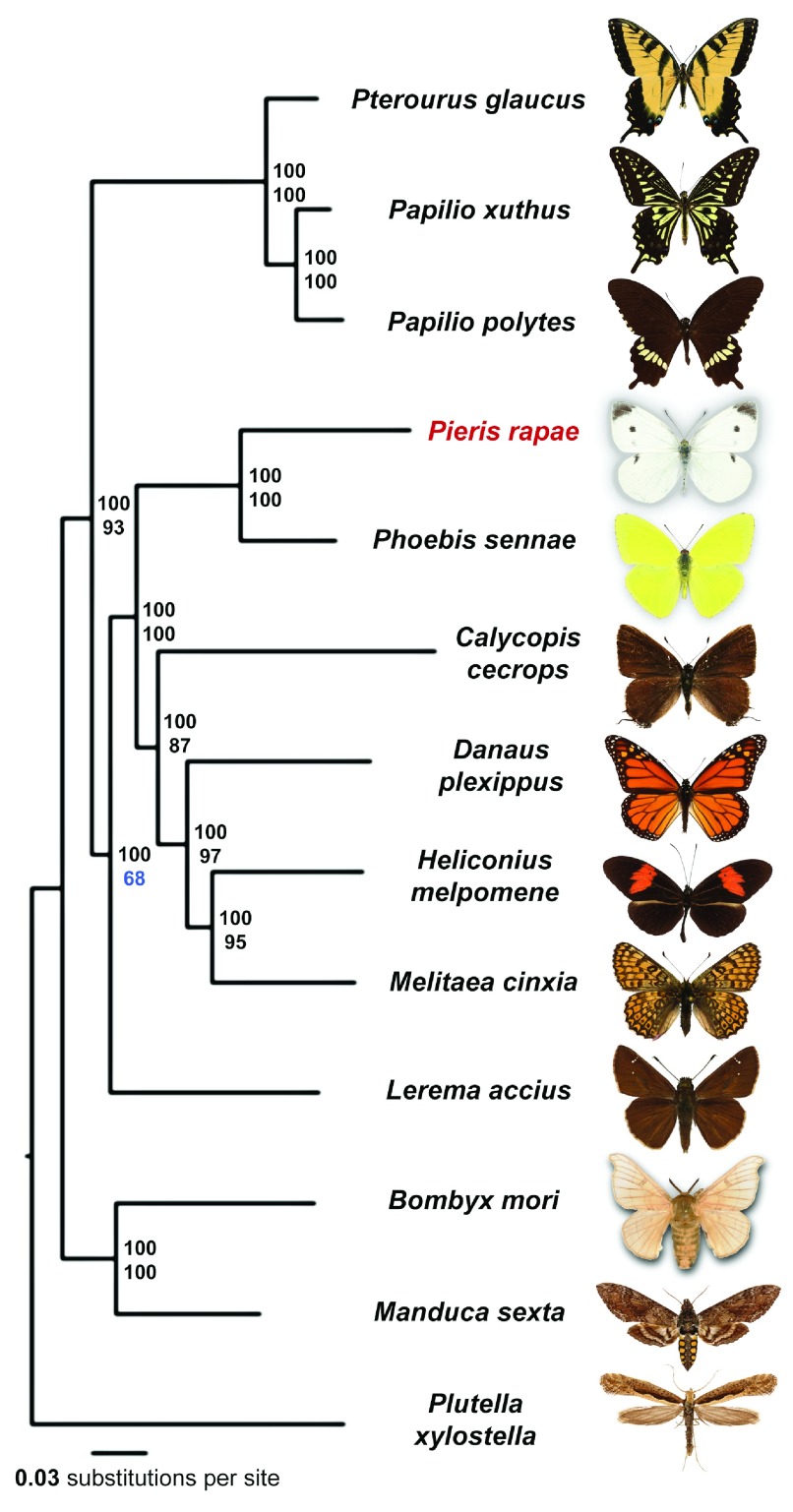
Phylogenetic tree of the Lepidoptera species with complete genome sequences. Majority-rule consensus tree of the maximal likelihood trees constructed by RAxML on the concatenated alignment of universal single-copy orthologous proteins. Numbers by the nodes refer to bootstrap percentages. The numbers above are obtained from complete alignments, the number below are obtained on 1% of the dataset.

We identified orthologous proteins encoded by 13 Lepidoptera genomes ( *Plutella xylostella*, *Bombyx mori*, *Manduca sexta*, *Lerema accius*, *Papilio glaucus*, *Papilio polytes*, *Papilio xuthus*, *Phoebis sennae*, *Melitaea cinxia*, *Heliconius melpomene*, *Danaus plexippus*, *Calycopis cecrops* and *Pieris rapae*) and detected 4906 universal orthologous groups, from which 1845 groups consist of a single-copy gene in each of the species. A phylogenetic tree built from the concatenated alignment of the single-copy orthologs using RAxML places *Pieris* as the sister to *Phoebis* ( Figure 2), the only other member of the Pieridae family with sequenced genome. Our analysis places Papilionidae as a sister to all other butterflies, including skippers (Hesperiidae). Such placement contradicts morphology-based phylogeny, but is reproduced in all maximum-likelihood and Bayesian trees published recently ^26, 28^.

All nodes received 100% bootstrap support when the alignment of all single-copy orthologs was used. However, since bootstrap only measures internal consistency of phylogenetic signal in the alignment, very large datasets will almost always result in 100% support, even if the tree is incorrect and biased by various factors such as nucleotide composition and long branch attraction. To find the weakest nodes, we reduced the amount of data by randomly splitting the concatenated alignment of all single-copy orthologs into 100 alignments (about 3088 positions in each alignment). The consensus tree based on these alignments revealed that the node referring to relative position of skippers and swallowtails shows the lowest support (68%) compared to other nodes, and their evolutionary history remains to be further investigated when better taxon sampling by complete genomes is achieved.

### Anti-cancer protein pierisin

We identified 8 copies of the pierisin gene ( Supplementary Table S3A), while only 2 copies were previously reported from *Pieris rapae* (GenBank) ^14, 15^. At least 7 pierisin copies are likely expressed, as their partial sequences are present in the RNA-seq data from adult. The pierisin protein resembles a classic bacterial AB-toxin, with an enzymatically active A domain toxin that is delivered across the eukaryotic membrane through interaction with receptors on the cell surface by the B domain. Pierisin is closely related to the bacterial mosquitocidal toxin MTX NAD(+)-dependent ADP-ribosyltransferase for which the crystal structure is known ^29^, with the closest pierisin sequence Pra57.2 having 32.56% identity to the structure sequence represented by the MTX holotoxin (PDB 2vse). The pierisin toxin transfers an ADP-ribosyl moiety to 2'-deoxyguanosine residues in DNA ^30^, while the ricin domains mediate interactions with neutral glycosphingolipid receptors, globotriaosylceramide (Gb3), and globotetraosylceramide (Gb4) ^31^. The toxin is thought to serve as a defense factor against parasitization by wasps ^12^, but also induces apoptosis in cancer cell lines ^10, 11, 32^.

**Figure 3.**
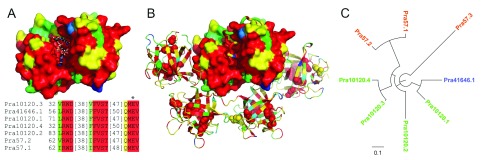
Pierisin conservation mapping to structure homolog and phylogenetic tree of the ricin domains. An alignment of the MTX holotoxin (PDB 2vse) sequence with the *Pieris rapae* pierisins was used to map sequence conservations calculated for the pierisin sequences. Conservations were colored in rainbow from blue (variable) to red (conserved). ( **A**) The N-terminal ADP-ribosylation toxin domain (shown in surface representation) of the MTX holotoxin structure was superimposed with the cholera ADP-ribosylation toxin bound to its NAD+ substrate (shown in ball and stick) to highlight the NAD+ binding pocket. An alignment of residues that contribute to the binding pocket are depicted below the structure, highlighted according to conservation, with the catalytic E marked by an asterisk. ( **B**) The N-terminal ADP-ribosylation toxin domain (shown in surface representation) of the MTX holotoxin is inhibited by a conserved inhibitory linker region (shown in tube) that blocks the substrate binding pocket. The C-terminal ricin-like domains of the holotoxin are depicted in cartoon, with corresponding sidechains of QxW motifs depicted in sphere. ( **C**) Phylogenetic tree of ricin domains in 8 pierisins from *Pieris rapae*.

Seven copies of pierisin encoded by the *Pieris rapae* genome include an N-terminal ADP-ribosylation toxin followed by an inhibitory linker and four ricin domains. Mapping the *Pieris rapae* pierisin sequence conservations (in rainbow from conserved red to variable blue) to the MTX holotoxin structure revealed a strict conservation of the active site and residues surrounding the NAD-binding site ( Figure 3A, NAD in ball and stick), as well as conservation of the inhibitory linker in the region that replaces NAD ( Figure 3B, linker in tube). The receptor-interacting ricin domains include QxW motifs that contribute to cytotoxicity ( Figure 3B, spheres), and display relatively lower overall conservation than the catalytic domain. Thus, the receptor-interacting function might be diverging across the different copies of the gene, potentially allowing broader receptor specificity.

One copy of pierisin (Pra57.3) lacks the N-terminal ADP-ribosylation domain, and is composed of four ricin domains following an N-terminal signal peptide, as validated by both the assembled genome and *de novo* assembled transcripts. In addition, the phylogenetic tree of the ricin domains in the eight copies of pierisin places this protein on the longest branch, suggesting that it has undergone rapid divergence from other pierisins and could have adopted a different function. Lacking the toxin domain, Pra57.3 may aid others toxins in entering the cells. Alternatively, it may be able to bind to the neutral glycosphingolipid receptors in the *Pieris*, and protect its own cells against other pierisins with the toxic ADP-ribosylation domains.

### Detoxifying nitrile-specifier proteins

During feeding, the cabbage white butterfly larvae possess the ability to counteract toxic secondary metabolites produced by the food plant glucosinolate–myrosinase major chemical defense system. The hydrolysis reaction of plant myrosinase, which normally produces toxic isothiocyanates, is redirected to the production of nitriles in the presence of the larval gut nitrile-specifier protein (NSP) ^13^. The exact role of NSP in nitrile production is debatable, the protein could either serve as an enzyme catalyzing the formation of nitriles from the aglycone intermediate or as an allosteric cofactor for myrosinase ^13, 33^. The detoxifying NSP protein belongs to an insect-specific gene family consisting of variable tandem repeating units termed insect allergen-related repeats. While other Lepidoptera genomes appear to have no NSP genes, the *Pieris* genome encodes two copies of the NSPs ( Supplementary Table S3B), each containing three copies of the insect allergen-related repeat domain ^34^.

Recently, a crystal structure of an insect allergen-related repeat domain from cockroach revealed a novel fold of twelve alpha-helices (two 6 helical repeating units) encapsulating a large hydrophobic cavity. While the sequence identity between the allergen structure and each of the three *Pieris* NSP domains is relatively low (~ 20% to each), their sequences can be confidently mapped to the known structure for functional inference. The cockroach allergen repeat cavity binds phospholipids such as phosphatidylethanolamine and phosphatidylglycerol when expressed in bacteria; and phosphatidylinositol (PI), phosphatidylserine and phosphatidylcholine when expressed in yeast. Alternately, the allergen purified from cockroach bound nonphosphorylated fatty acids such as palmitate, stearate, and oleate ^35^, revealing a promiscuous binding capacity of the hydrophobic pocket. Such a promiscuous allergen binding activity might translate to the sequence-related NSP pockets, allowing binding of the various aglycone intermediates of the glucosinolate–myrosinase system.

**Figure 4.**
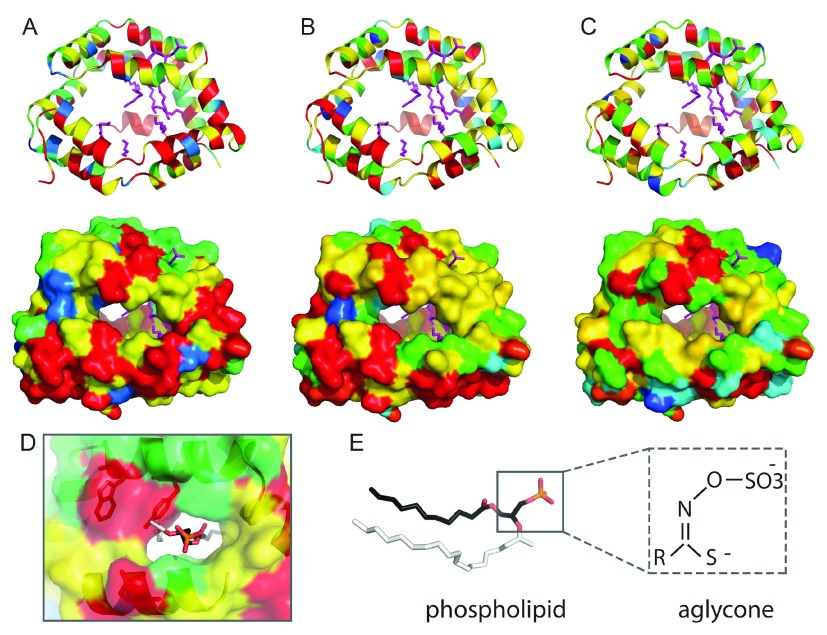
NSP family sequence conservation mapping to the insect allergen repeat structure. Residue conservation is colored from red (invariant) to blue (variable). The NSP N-terminal ( **A**), middle ( **B**), and C-terminal ( **C**) domain repeats are represented in ribbon (upper panels) and surface (lower panels). Lipids from the insect allergen structure (4jrb) are in magenta sticks. ( **D**) Zoom into the phospholipid binding site (N-terminal domain), with the head group colored by atom: P (orange), O (red), and C (black). The larger side group of the phospholipid ligand (white) is not compatable with the NSP YxxxW _187_ motif (shown in stick). ( **E**) Comparison of phospholipid ligand (stick representation) with aglycone, with similar atom backbone orientations boxed. Sequence conservations were calculated using Al2CO ^72^ from an alignment of the following: Pieridae NSP1 and NSP2, together with AAR84202.1, ABY88944.1, ABX39547.1, ABX39554.1, ABY88945.1, ABX39555.1, ABX39546.1, ABX39549.1, ABX39537.1, ABX39552.1, ABX39553.1 from the NCBI Non-redundant protein database.

Mapping the NSP-related protein sequences conservations to the allergen structure highlights invariant residues that both line the hydrophobic cavity of each domain, connect the repeating units, and connect adjacent *α*-helices of the repeat ( Figure 4, conserved residues colored red). The hydrophobic nature of the binding cavity is preserved in the NSP sequences, including numerous invariant hydrophobic residues that likely contribute to function. Conserved NSP residues also reside near the PO4 group of the phospholipid binding site ( Figure 4D), including a YxxxW motif found in each repeat that should restrict the site to accommodate smaller ligands. In fact, the aglycone intermediate SO4 group and adjacent backbone atoms could mimic the PO4 in phospholipid ( Figure 4E).

Alternately, the positions of invariant polar residues are limited to those that contribute to *α*-helical interactions, to the linker regions that do not line the hydrophobic cavity, or to insertions not present in the template allergen-repeat structure. While an active site could potentially form between repeating domains of the NSP structure, no obvious clusters of catalytic residues could be mapped to the individual cavities of any of the domain repeats present in NSP. Potentially, the NSP cavities could accommodate binding the various aglycone intermediates produced by myrosinase, allowing time for spontaneous conversion to simple nitriles in the low pH of the gut. Thus, the NSP binding cavity could act in a pseudo-enzymatic capacity, without traditional catalytic residues mediating chemistry.

### Inferring the population history from the SNP distribution pattern

While the *Pieris rapae* genome is very heterozygous at 1.5%, the distribution of these SNPs in the genome is highly non-random. The histogram of SNP fraction in 1000 bp genomic windows for both *Pieris rapae* and *Papilio glaucus* ( *Pgl*) is shown in Figure 4A. Since the reads from the highly heterozygous regions in the genome may not map well to the reference genome, such regions usually show lower-than-expected coverage and may hinder the detection of heterozygous positions. Therefore, in the analysis of both *Pgl* and *Pra* genomes, we focused on the genomic regions with coverage that are expected for a diploid genome. Compared to *Pgl*, the *Pra* genome contains a much higher fraction of homozygous (SNP-free) regions ( Figure 4B). This difference cannot be simply explained by the relatively low heterozygosity of *Pra* (1.5% for *Pra* and 2.3% for *Pgl*), because the probability of observing SNP-free segments longer than 500 bp is below 1% for genome of this size having 1.5% of heterozygosity ( Figure 4C).

The *Pra* genome assembly contains a large portion (18.3% of the total length) of SNP-free segments that are at least 1,000 bp. The average coverage of the SNP-free segments by the reads is 87 fold, which is higher than the average coverage of all the segments under study (coverage: 84 fold). Therefore, the lack of heterozygous positions does not arise from the failure of mapping reads from one haplotype to the reference genome which represents another haplotype in the highly heterozygous region. The *Pgl* genome contains only 1.55% long (>= 1000 bp) SNP-free segments, which also support that the large portion of SNP-free segments in the *Pra* genome is not an artifact.

The median length of these segments is 38,000 bp, and the longest SNP-free region in the *P. rapae* draft genome is 339,000 bp. The presence of such high proportion of SNP-free segments indicates that this *Pra* specimen inherited a large proportion of identical alleles from its parents. Two scenarios could explain this: (1) this specimen is a result of recent inbreeding between brothers and sisters or between cousins (2) the population started from a very small number of individuals or has been through very severe bottlenecks and therefore the genetic diversity within the population is low. In order to distinguish between these two scenarios, we simulated them.

Inbreeding between brother and sister would result in the presence of ~25% long homozygous segments, and this ratio goes down to 6.3% when the parents are cousins ( Figure 6A). Inbreeding between half-blooded brother and sister from the same father (or mother) and whose mothers are sisters would result in 18.6% of homozygous segments. However, inbreeding between very close relatives would result in a very high median lengths of the SNP-free segments ( Figure 6B), even if we assumed a very high recombination rate, 10 cM/Mb ^36^. The median length of SNP-free segments in this scenario is still above 200,000 bp, which is much higher than the observed value, 38,000 bp. Therefore, inbreeding between close relatives cannot explain the observed SNP pattern.

**Figure 5.**
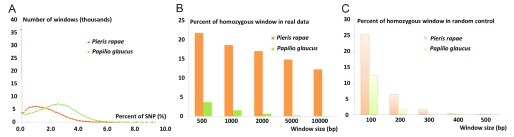
Comparison of SNP patterns in *Pieris rapae* ( *Pra*) and *Papilio glaucus* ( *Pgl*) *.* ( **A**) Histogram of SNP rates in 1000 bp windows from the *Pra* (red orange curve) and *Pgl* (green curve) genome. ( **B**) The fraction of SNP-free long genomic windows in the *Pra* (orange bars) and *Pgl* (green bars) genomes. *Pra* genome has a much larger fraction of SNP-free windows than *Pgl*, especially when the window size goes beyond 1,000 bp. ( **C**) The fraction of SNP-free genomic windows in *Pra* (light orange bars) and *Pgl* (light green bars) if the SNPs are distributed randomly. The fraction of such windows goes down to 0 when the window size is equal or bigger than 1000 bp.

**Figure 6.**
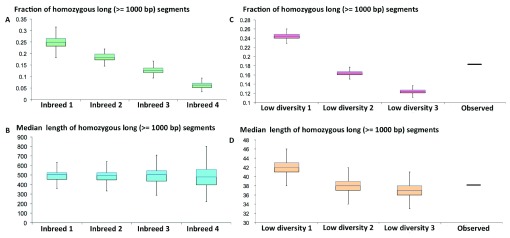
The fraction and median length of SNP-free segments observed in the genome supports the hypothesis that the population in America started with few individuals. ( **A**) The fraction and ( **B**) median length of SNP-free segments in the offspring of inbreeding between very close relatives. Inbreed 1: inbreeding between brother and sister; Inbreed 2: inbreeding between half-blooded brother and sister with common father (or mother) whose mothers (or fathers) are also sisters (or brothers) of each other. Inbreed 3: inbreeding between half-blooded brohter and sister with common father (or mother) whose mothers (or fathers) are not related. Inbreed 4: inbreeding between cousins. ( **C**) The fraction and ( **D**) median length of SNP-free segments in an individual from a *in silico* simulated population with low genetic diversity. Low divesity 1, 2, and 3: populations start from 2, 3, and 4 individuals, respectively. 500 generations with an effective population size of 50,000 were simulated. The recombination and mutation rates are 5 cM/Mb and 2.5e-3/Mb per generation.

The observed pattern of SNP-free segments agrees very well with the second scenario, i.e., the genetic diversity in the population is low, because the population started from very small number of individuals or has undergone very severe bottlenecks. The observed fraction and median lengths of the long SNP-free segments agrees very well with the simulated data assuming that the population started with 3 individuals (could be one female carrying spermatophores of two males) and has been developing for about 500 generations ( Figure 6C, D). This supports the hypothesis that *Pieris rapae* came to America in 19 ^th^ century and the population started from very few individuals introduced by human activity. It cannot be excluded that the population started with a larger number of introduced individuals, but the genetic diversity was reduced due to severe bottlenecks, possibly early on, so only the progeny of one or two females gave rise to American populations of *Pieris rapae*. However, as a widely spread butterfly species over all different habitats that is somewhat resistant to parasite and toxins in plant, bottlenecks in the later stage of population history is not very likely.

## Materials and methods

### Library preparation and sequencing

Major in-house scripts, archived at the time of publication Please see README.txt for a description of the files. Click here for additional data file. Copyright: © 2016 Shen J et al. 2016 Data associated with the article are available under the terms of the Creative Commons Zero "No rights reserved" data waiver (CC0 1.0 Public domain dedication).

We removed and preserved the wings and genitalia of three freshly caught *Pieris rapae* specimens (NVG-3537 female, NVG-3842 and NVG-4113 males from USA: Texas: Dallas Co., Dallas, GPS 32.90516, -96.81546, collected on 5-Jun-2015, 30-Jun-2015, 17-Jul-2015, respectively), while the rest of the bodies were stored in *RNAlater* solution (Life Technologies Corporation, Grand Island, NY USA). Wings and genitalia of these specimens will be deposited in the National Museum of Natural History, Smithsonian Institution, Washington, DC, USA (USNM).

We used specimens NVG-3842 and NVG-4113 for sequencing and assembly the reference genome. We extracted genomic DNA from the tissue with the ChargeSwitch gDNA mini tissue kit (Invitrogen, Waltham, MA USA). 250 bp and 500 bp paired-end libraries were prepared using genomic DNA from specimen NVG-3842 with enzymes from NEBNext Modules (New England Biolabs Inc., Ipswich, MA USA) and following the Illumina TruSeq DNA sample preparation guide http://prodata.swmed.edu/LepDB/Protocol/illumina_Paired-End_Sample_Preparation_Guide.pdf. 2 kb, 6 kb and 15 kb mate pair libraries were prepared using genomic DNA from both NVG-3842 and NVG-4113 with a protocol similar to previously published Cre-Lox-based method ^37^. For the 250 bp, 500 bp, 2 kbp, 6 kbp and 15 kbp libraries, approximately 250 ng, 250 ng, 0.96 μg, 1.92 μg and 2.87 μg of isolated DNA were used, respectively. We quantified the amount of DNA from all the libraries with the KAPA Library Quantification Kit (Kapa Biosystems, Inc., Wilmington, MA USA), and mixed 250 bp, 500 bp, 2 kbp, 6 kbp, 15 kbp libraries at relative molar concentrations of 40:20:8:4:3. The mixed library was sequenced with PE-150 bp run using 64% of a single Illumina lane on HiSeq 2500 at UT Southwestern Medical Center Genomics and Microarray Core Facility.

Part of specimen NVG-3537 was used to extract RNA using QIAGEN RNeasy Mini Kit (QIAGEN Inc., Valencia, CA USA). We further isolated mRNA using NEBNext Poly(A) mRNA Magnetic Isolation Module (New England Biolabs Inc., Ipswich, MA USA). RNA-seq libraries were prepared with NEBNext Ultra Directional RNA Library Prep Kit (New England Biolabs Inc., Ipswich, MA USA) for Illumina following manufacturer’s protocol. The RNA-seq library was sequenced with PE-150 bp run using 9% of an Illumina lane. The sequencing reads of all these libraries were deposited in the NCBI SRA database under accession SRP073457.

### Genome and transcriptome assembly

We removed sequence reads that did not pass the purity filter and classified the remaining reads according to their TruSeq adapter indices to get individual sequencing libraries. Mate pair libraries were processed by the Delox script ^37^ to remove the loxP sequences and to separate true mate pair from paired-end reads. All reads were processed by mirabait ^38^ v4.0.2 to remove contamination from the TruSeq adapters, an in-house script to remove low quality portions (quality score < 20) at the ends of both reads, JELLYFISH ^39^ v2.2.3 to obtain k-mer frequencies in all the libraries, and QUAKE ^40^ v0.3.5 to correct sequencing errors. The data processing resulted in seven libraries that were supplied to Platanus ^41^ v1.2.4 for genome assembly: 250 bp and 500 bp paired-end libraries, 2 kbp, 6kbp, 15k bp true mate pair libraries, a library containing all the paired-end reads from the mate pair libraries, and a single-end library containing all reads whose pairs were removed in the process.

We mapped these reads to the initial assembly with Bowtie2 ^42^ v2.2.3 and calculated the coverage of each scaffold with the help of SAMtools ^43^ v1.0. Many short scaffolds in the assembly showed coverage that was about half of the expected value; they likely came from highly heterozygous regions that were not merged to the equivalent segments in the homologous chromosomes. We removed them if they could be fully aligned to another significantly less covered region (coverage > 90% and uncovered region < 500 bp) in a longer scaffold with high sequence identity (>95%). Similar problems occurred in the *Heliconius melpomene*, *Pterourus glaucus* and *Lerema accius* genome projects, and similar strategies were used to improve the assemblies ^19, 24, 26^.

The RNA-seq reads were processed using a similar procedure as the genomic DNA reads to remove contamination from TruSeq adapters and the low quality portion of the reads. Afterwards, we applied three methods to assemble the transcriptomes: (1) *de novo* assembly by Trinity ^44^ v2.0.6, (2) reference-based assembly by TopHat ^45^ v2.0.10 and Cufflinks ^46^ v2.2.1, and (3) reference-guided assembly by Trinity v2.0.6. The results from all three methods were then integrated by Program to Assemble Spliced Alignment (PASA) ^47^ v2.0.2.

### Identification of repeats and gene annotation

Two approaches were used to identify repeats in the genome: the RepeatModeler ^48^ v1.0.7 pipeline and in-house scripts that extracted regions with coverage 3 times higher than expected. These repeats were submitted to the CENSOR ^49^ server to assign them to the repeat classification hierarchy. The species-specific repeat library and all repeats classified in RepBase ^50^ v18.12 were used to mask repeats in the genome by RepeatMasker ^51^ v4.0.3.

We obtained two sets of transcript-based annotations from two pipelines: TopHat followed by Cufflinks and Trinity followed by PASA. In addition, we obtained eight sets of homology-based annotations by aligning protein sets from *Drosophila melanogaster* ^52^ and seven published Lepidoptera genomes ( *Bombyx mori*, *Lerema accius*, *Papilio polytes*, *Papilio glaucus*, *Papilio xuthus*, *Heliconius melpomene*, and *Danaus plexippus*) to the *Pra* genome with exonerate ^53^ v2.2.0. Proteins from insects in the entire UniRef90 ^54^ database were used to generate another set of gene predictions by genblastG ^55^ v1.38. We manually curated and selected 1256 confident gene models by integrating the evidence from transcripts and homologs to train *de novo* gene predictors: AUGUSTUS ^56^ v3.1, SNAP ^57^ and GlimmerHMM ^58^ v3.0.3. These trained predictors, the self-trained Genemark ^59^ v2.3e and a consensus-based pipeline Maker ^60^ v2.31.8, were used to generate another five sets of gene models. Transcript-based and homology-based annotations were supplied to AUGUSTUS, SNAP and Maker to boost their performance. In total, we generated 16 sets of gene predictions and integrated them with EvidenceModeller ^47^ v1.1.1 to generate the final gene models.

We predicted the function of *Pra* proteins by transferring annotations and GO-terms from the closest BLAST ^61^ v2.2.30 hits (E-value < 10 ^-5^) in both the Swissprot ^62^ database and Flybase ^63^. Finally, we performed InterproScan ^64^ v5.17-56.0 to identify conserved protein domains and functional motifs, to predict coiled coils, transmembrane helices and signal peptides, to detect homologous 3D structures, to assign proteins to protein families and to map them to metabolic pathways.

### Identification of orthologous proteins, analysis of unique genes for *Pieris rapae*, and phylogenetic tree reconstruction

We identified the orthologous groups from 13 Lepidoptera genomes using OrthoMCL ^65^ v2.0.9. The orthologous groups that contain only *Pieris* proteins were further investigated. Starting from these *Pieris* sequences, we attempted to identify their orthologs in other Lepidoptera genomes using reciprocal BLAST. Potential orthologs encoded by the genome but missed in the protein sets were predicted with the help of genblastG. Two groups of proteins, i.e. the pierisins and nitrile-specifier proteins discussed above turned out to be unique for *Pieris*. We manually curated the sequences for proteins in these two groups and submitted them to MESSA ^66^ to perform secondary structure and disordered region prediction, domain identification and 3D structure prediction. We aligned the pierisin sequences using MAFFT v7.237 and built their evolutionary tree with RAxML ^67^ v8.2.3 and visualized them in FigTree v1.4.2.

1845 orthologous groups consisted of single-copy genes from every species, and they were used for phylogenetic analysis. An alignment was built for each universal single-copy orthologous group using both global sequence aligner MAFFT ^68^ and local sequence aligner BLASTP. Positions that were consistently aligned by both aligners were extracted from each individual alignment and concatenated to obtain an alignment containing 308,750 positions. The concatenated alignment was used to obtain a phylogenetic tree using RAxML. Bootstrap resampling of the aligned positions was performed to assign the confidence level of each node in the tree. In addition, in order to detect the weakest nodes in the tree, we reduced the amount of data by randomly splitting the concatenated alignment into 100 alignments (about 3,088 positions in each alignment) and applied RAxML to each alignment. We obtained a 50% majority rule consensus tree and assigned confidence level to each node based on the percent of individual trees supporting this node.

### Conservation mapping of NSP and pierisin

NSP family sequences were collected using BLAST (PMID: 9254694) of the nr database with NSP1 as a query (default settings), keeping subject sequences with over 90% coverage. Conservations were calculated using Al2CO (PMID: 11524371) from a MAFFT (PMID: 24170399) alignment of the following: Pieridae NSP1 and NSP2, together with AAR84202.1, ABY88944.1, ABX39547.1, ABX39554.1, ABY88945.1, ABX39555.1, ABX39546.1, ABX39549.1, ABX39537.1, ABX39552.1, ABX39553.1. The NSP family includes three copies of an Insect allergen related repeat domain, which has a structure representative of the cockroach allergen Bla G 1 (PDB: 4jrp). The 4jrp sequence was aligned with each of the three repeat domains in the NSP family using PSI-BLAST (PMID: 9254694) and HHPRED (PMID: 9626712) alignments as guides. Positional conservations for each domain were mapped to the B-factor column of the 4jrp structure coordinates with AL2CO (PMID: 11524371), and displayed with rainbow color scale (from blue variable to red conserved) using PyMOL Molecular Graphics System. Eight copies of pierisin from the sequenced genome were aligned as above with the related MTX holotoxin sequence HHPRED (PDB: 2vse), calculating and displaying positional conservations as above.

### Analysis of the SNP patterns in *Pieris rapae*

We analyzed the SNPs in *Pra* and *Papilio glaucus* ( *Pgl*) genomes using the same protocol, in which we mapped each read to the genomes and detected SNPs using the Genome Analysis Toolkit ^69^ v3.5. The distribution of genome coverage by the reads in 100 bp windows was plotted. For both *Pra* and *Pgl* genomes, this distribution shows two peaks. In addition to the main peak centered at the expected coverage for a diploid genome, there is an additional peak to the left that corresponds to highly divergent regions between the two homologous chromosomes. Owing to this sequence divergence, only the reads corresponding to the sequence of one of the homologous chromosomes can be mapped, which results in the lower-than-expected coverage. To analyze the distribution of SNPs, we used the regions whose coverage by the reads falls within the diploid peak.

We calculated the total number of positions with SNPs in such regions and simulated random distribution of these SNPs. The simulated distributions were used as controls. For the random control, experimental data, and the simulated genomes discussed below, we divided the scaffolds into 100, 200, 300, 400, 500, 1000, 2000, 5000, and 10000 bp windows (segments less than the window length at the ends of scaffolds are discarded), respectively, and calculated the presence of SNP-free windows. We concatenated neighboring SNP-free regions to obtain the longest SNP-free segments, and calculated the median length of these SNP-free segments.

### Simulation of recent inbreeding and evolutionary history of the *Pieris rapae* population in America

We simulated *Pieris rapae* haplotypes by randomly introducing SNPs to the *Pra* reference genome, and the frequency of SNPs was set to be half of the frequency of heterozygous positions in the sequenced *Pra* individual (i.e., 0.7%). Two simulated haplotypes were randomly paired to represent another simulated *Pieris rapae* individual, and the rate of heterozygous positions in the simulated individuals would be comparable to that observed in the sequenced specimen. To simulate the mating between two individuals, we assumed the two haplotypes of each individual could recombine at a certain rate (recombination rate) and generate a new haplotype that is inherited to the offspring.

The recombination rates of insects are rather variable, and the recombination rates for *Bombyx mori*, *Heliconius melpomene* and *Heliconius erato* are estimated to be 2.6, 5.5 and 6.1, respectively ^36^. Therefore, we estimated the recombination rate for *Pieris rapae* to range between 1cM/Mb and 10cM/Mb per generation. To simulate recent inbreeding, we randomly select a recombination rate within this range. The mutations in this process are not introduced because the per generation mutation rate for butterflies are expected to be in the magnitude of 1e-9 mutation per base pair ^70^, much lower than the existing variation between haplotypes. We simulated three scenarios of inbreeding: (1) between brother and sister (2) between cousins and (3) between half-blooded brother and sister.

To simulate the evolution of *Pieris rapae* population, we assumed the population started from a certain number of individuals (2, 3 and 4). Several parameters would affect the population evolution, i.e., the number of generations since the species invaded America, the recombination rate, the mutation rate, and the effective population size. *Pieris rapae* was suggested to invade America in the second half of 19 century, and has 3–6 generations per year. Therefore, we assumed the number of generations to be 500. Based on the known values for other Lepidoptera species, we assumed the recombination rate to be 5cM/Mb and the mutation rate to be 2.5e-9. In the initial generations, the effective population size is mainly limited by the population size, and the population may undergo exponential growth. We assumed an exponential growth of the effective population size at rate of 10 fold per generation (each pair produce 20 off-springs). Later on, the population may reach its stationary phase, and the effective population size will be limited by the population structure and will not keep increasing. The effective population size of insects usually ranges between 1e5 and 1e6 ^71^, and we assumed the effective population size to be 5e5 after the initial exponential growth phase.

## Data availability

The data referenced by this article are under copyright with the following copyright statement: Copyright: © 2016 Shen J et al.

Data associated with the article are available under the terms of the Creative Commons Zero "No rights reserved" data waiver (CC0 1.0 Public domain dedication). 

Sequencing reads were deposited in the NCBI SRA database under accession number SRP073457. The genome sequence was deposited at DDBJ/EMBL/GenBank under accession number LWME00000000.

Major in-house scripts and intermediate results are available at http://prodata.swmed.edu/LepDB/.

Archived scripts at the time of publication: 10.5256/f1000research.9765.d140486 ^73^

Please see README.txt for a description of the files.

## Supplementary material

**Supplementary Table S1. Quality and composition of Lepidoptera genomes, related to Table 1.**

.

**Supplementary Table S2. Statistics for sequencing data and data processing related to experimental procedures and genome annotation.**

.

**Supplementary Table S3. Protein sequences of pierisins and nitrile-specifier.**
