# Supplementary material for: Complete genome of Pieris rapae, a resilient alien, a cabbage pest, and a source of anti-cancer proteins
Source: F1000Res. 2016 Nov 3;5:2631. [Version 1] doi: 10.12688/f1000research.9765.1 (PMC5247789; doi:10.12688/f1000research.9765.1)
Supplement: Peer review history

# Peer review: Referee response for version 1

**DOI:** 10.5256/f1000research.10527.r19401

**Published:** 2017-01-17

**Authors:** Andrei Sourakov

**Affiliations:** 1 McGuire Center for Lepidoptera and Biodiversity, Florida Museum of Natural History, University of Florida, Gainesville, FL, USA

This paper is an important contribution to genomics. The choice of the cabbage white butterfly for this study is especially fitting, as chemical interactions between plants and herbivores have been recently studied on molecular level using this species and its relatives, with some very interesting insights into evolution of detoxification abilities in insects.  The paper also addresses several additional questions from phylogenetics of Lepidoptera to the origins of the invasive exotic pest in North America. The authors provide creative solution for distinguishing between modern inbreeding and historical population bottleneck and interesting observations concerning recreation of evolutionary history using full-genome information. The potentially applied aspects of the paper - presence in the model organism of protein with anti-cancer properties and mapping of this protein – make this study a valuable foundation for future medical research.

 I am also attaching the pdf of the article with minor suggestions concerning grammar/wording.

I have read this submission. I believe that I have an appropriate level of expertise to confirm that it is of an acceptable scientific standard.

# Peer review: Referee response for version 1

**DOI:** 10.5256/f1000research.10527.r19231

**Published:** 2017-01-12

**Authors:** James Mallet

**Affiliations:** 1 Department of Organismic and Evolutionary Biology, Harvard University, Cambridge, MA, USA

**Title and abstract:** Appropriate, and suitable summary

**Article content: **This is another of many butterfly genome assemblies pioneered by the Nick Grishin group based on new short-read sequencing technology.  It will form a useful contribution, and the fact that the butterfly is a major crop pest is a good justification for sequencing this particular species. Also the authors mention some novel biochemical pathways the insects use, hitherto not found in any other species. One set of proteins (pierisins) may be useful in inducing apoptosis and has already been suggested as an anti-cancer treatment – the discovery of more of these proteins in the current genome and transcriptomics work may be important in medicine. In addition, the "nitrile specifier proteins" are involved in inactivation of plant defences, and are another novel protein found in this species only. I'm not an expert on structural biology, but the authors apparently are, and so I'd defer to their knowledge of this area.

 Pieris rapae is introduced from the old world, and so it might be expected to have undergone a population bottleneck or founder event. There was an intriguing pattern of homozygous patches in the genome that were suggested to be consistent with the hypothesis of such a bottleneck from a single female or maybe a couple of individuals having been introduced to North America in the last century. However, the simulation approach based on only a couple of genomes made this aspect of the study rather weak, especially since a major part of the article was devoted to these results. If the authors were really interested in this topic, they might have sampled resequence data more broadly, especially in the putative native range (presumably Europe?), as well as across North America. There are such studies from way back, for instance I quickly found this old study by googling, and the enzyme (protein) heterozygosities therein do not look particularly low by butterfly standards: Vawter, A.T., & Brussard, P.F. 1984 ^1^. There's also an intriguing difference between Southern and Western populations compared to Northeastern populations which may suggest greater heterozygosity in areas with climate more similar to Northern Europe. In other words, the long discussion in the paper about the heterozygosity and putative bottleneck in this study are misplaced, seem to ignore prior work, and seek to re-invent population genetic analyses rather than employing a more direct, standard approach to studying the putative bottleneck. I'd suggest greatly shortening this section, or doing the extra work to ensure that there are more comparisons with resequenced individuals, and more attention paid to prior work in this area.

 How confident are the authors of their identification of repeats? I was a little unclear about the methods used here, but they appear to be using the assembly itself to identify repeats. This has very well known weaknesses in next-gen genome sequence assemblies. I recently examined the major Lepidoptera genomes for the presence of ribosomal DNA repeats, and found that none of these next-gen assembly genomes could not assemble the rDNA genes in any semblance of the way they ought to be. I think it's an assembly problem.

**Conclusions:** see above

**Data:** provision is adequate and standard for genome sequences.

I have read this submission. I believe that I have an appropriate level of expertise to confirm that it is of an acceptable scientific standard.

## References

[1] VawterATBrussardPF: Allozyme variation in a colonizing species: the cabbage butterfly Pieris rapae (Pieridae). *Journal of Research on the Lepidoptera*.1984;22:204-216
